# Levetiracetam attenuates hippocampal expression of synaptic plasticity-related immediate early and late response genes in amygdala-kindled rats

**DOI:** 10.1186/1471-2202-11-9

**Published:** 2010-01-27

**Authors:** Kenneth V Christensen, Henrik Leffers, William P Watson, Connie Sánchez, Pekka Kallunki, Jan Egebjerg

**Affiliations:** 1 Dept. of Molecular Biology, Discovery Biology Research, H. Lundbeck A/S, DK-2500 Valby, Denmark; 2 University Dept. of Growth and Reproduction, Rigshospitalet, DK-2100 Copenhagen, Denmark; 3 Dept. of Neuropharmacology, H. Lundbeck A/S, DK-2500 Valby, Denmark; 4 Current address: Teva Pharmaceutical Ltd, The Gate House, Gatehouse Way, Aylesbury, UK; 5 Current address: Dept. of Neuroscience, Lundbeck Research Inc. USA, Paramus-NJ, USA

## Abstract

**Background:**

The amygdala-kindled rat is a model for human temporal lobe epilepsy and activity-dependent synaptic plasticity. Hippocampal RNA isolated from amygdala-kindled rats at different kindling stages was analyzed to identify kindling-induced genes. Furthermore, effects of the anti-epileptic drug levetiracetam on kindling-induced gene expression were examined.

**Results:**

Cyclooxygenase-2 (Cox-2), Protocadherin-8 (Pcdh8) and TGF-beta-inducible early response gene-1 (TIEG1) were identified and verified as differentially expressed transcripts in the hippocampus of kindled rats by *in situ *hybridization and quantitative RT-PCR. In addition, we identified a panel of 16 additional transcripts which included Arc, Egr3/Pilot, Homer1a, Ania-3, MMP9, Narp, c-fos, NGF, BDNF, NT-3, Synaptopodin, Pim1 kinase, TNF-α, RGS2, Egr2/krox-20 and β-A activin that were differentially expressed in the hippocampus of amygdala-kindled rats. The list consists of many synaptic plasticity-related immediate early genes (IEGs) as well as some late response genes encoding transcription factors, neurotrophic factors and proteins that are known to regulate synaptic remodelling. In the hippocampus, induction of IEG expression was dependent on the afterdischarge (AD) duration. Levetiracetam, 40 mg/kg, suppressed the development of kindling measured as severity of seizures and AD duration. In addition, single animal profiling also showed that levetiracetam attenuated the observed kindling-induced IEG expression; an effect that paralleled the anti-epileptic effect of the drug on AD duration.

**Conclusions:**

The present study provides mRNA expression data that suggest that levetiracetam attenuates expression of genes known to regulate synaptic remodelling. In the kindled rat, levetiracetam does so by shortening the AD duration thereby reducing the seizure-induced changes in mRNA expression in the hippocampus.

## Background

Human temporal lobe epilepsy (TLE) is a neurological brain disorder affecting approximately 0.5% of the population [1]. There exist monogenetic forms of TLE. However, most forms are either symptomatic or idiopathic [2,3]. The symptomatic forms of human temporal lobe epilepsy results from various kinds of brain injury, e.g. stroke, ischemia, head trauma, infections, febrile seizures and brain surgery [4]. In these cases the epileptic process generally comprises three phases i) the initial precipitating insult ii) the latent epileptogenic phase and iii) epilepsy, i.e. recurrent complex partial seizures with secondary generalisation. Most of the current drug therapies only relieve the convulsive state whereas they have no clinically documented effect on the development of the epileptic condition [5]. TLE is also the most common form of treatment resistant epilepsy in adult epileptic patients. Here, the seizure activity typically originates in the hippocampal formation, which also exhibits neuropathological features such as hippocampal sclerosis associated with the TLE [6]. Ideally, animal models of epilepsies should share the characteristics of the acquired human disease, i.e. a) exhibit spontaneous seizures following the latent period, b) have a resembling pathophysiology which should include hippocampal sclerosis c) exhibit synaptic and neuronal reorganisation which should include mossy fiber sprouting and neurogenesis and finally, d) have a similar pharmacological profile [7]. The amygdala-kindling model initially characterized by Goddard and colleagues [8] comprises many if not all of these features of human TLE [5,8-10].

The newer anti-epileptic drug levetiracetam has shown anti-epileptic efficacy in the amygdala-kindled rat [11-13]. In addition, it reduces the frequency of spontaneous seizures after pilocarpine-induced status epilepticus [14]. Levetiracetam has proven efficacious as adjunctive therapy in patients with partial seizures [15] and in treating patients with idiopathic generalized epilepsy where therapy with other anti-epileptic drugs has failed [16]. It also reduces the frequency of interictal epileptiform discharges in patients with partial seizures [17]. In contrast, levetiracetam is devoid of anti-convulsant activity in maximal electroshock and pentylenetetrazole (PTZ) seizure tests [18,19]. Levetiracetam binding is highly enriched in cortical, hippocampal and cerebellar regions [20] and it can selectively bind to synaptic fractions purified from these regions [21]. The binding site for levetiracetam has been identified as the synaptic vesicle protein 2A (SV2A) [22]. It is expressed in synaptic vesicles [23] and although the exact function of SV2A has not yet been resolved the functional significance of SV2A is evident from studies showing that homozygous KO mice suffer from severe seizures and die postnatally [24,25]. Several studies have reported on late response gene expression profiling in rats with status epilepticus-induced spontaneous seizures [26-28]. However, based on the involvement of the hippocampal formation in human TLE we hypothesize that additional insights into the basic mechanisms and effects of levetiracetam treatment of human TLE might be achieved by also studying early gene expression changes in hippocampal tissue isolated from the amygdala-kindling rat model of human TLE. Thus, in the present study we aim at the following. Firstly, to identify differential expressed early genes (within 3 hours after the last seizure) by analysing gene expression changes in the hippocampus from amygdala-kindled rat as a function of kindling and secondly, examine the effect of levetiracetam on kindling-induced gene expression.

## Methods

### Animals

Male Wistar rats (Møllegård Breeding Center, Denmark) weighing approximately 300 g at the time of surgery were housed in plastic cages (35 × 30 × 12 cm) in groups of 2. Room temperature (21 ± 2°C), relative humidity (55 ± 5%), and air exchange (16 times per hour) were automatically controlled. The animals had free access to conventional laboratory chow and water and they were subjected to a normal light:dark cycle (light 06:00-18:00). All animal procedures were carried out in compliance with the EC Directive 86/609/EEC and with the Danish law regulating experiments on animals.

### Amygdala-kindling and levetiracetam treatment

Animals were anaesthetized with 2 ml/kg subcutaneously (s.c.) administered Hypnorm/Dormicum/H_2_O (1:1:2) v/v. Bipolar electrodes were stereotactically implanted into the basolateral amygdaloid nuclei of the right amygdala according to Paxinos' coordinates (DV -8.4, L -4.8 and AP -2.8 from bregma). One week after surgery afterdischarge (AD) threshold currents were determined by gradually increasing the stimulating current by 25 μA from 75 μA until an AD was evoked. Here, an AD was defined as a spike train with duration of at least 3 seconds. Subsequently, the rats were electrically stimulated every second weekday (Mondays, Wednesdays and Fridays; Weekends off) at the pre-determined AD threshold current. AD duration was measured by EEG and seizure severity (SS) was scored according to Racine's 5-stage behavioural scale for convulsive responses [29]. Seizure duration was scored as the duration of a convulsive seizure; however, this was only assessed for stage 5 animals. The animals were divided into kindled animals and non-kindled control animals. All animals had electrodes implanted in the basolateral amygdala. Prior to the experiment, the kindled animals were paired, with one vehicle treated and one levetiracetam treated rat in each kindling pair. The kindled animal pairs were stimulated 1 hour after either i.p. injection of vehicle or 40 mg/kg levetiracetam, respectively. During the experiment, animal pairs were sacrificed at different behavioural stages thus giving a total of 5 experimental groups (see Table 1): Group 1 consisted of operated but non-kindled rats and was sacrificed at the start of the experiment (n = 5); group 2, consisted of animals with 1-3 consecutive stage 3 seizures (n = 7); group 3, consisted of animals with one or two stage 5 seizures (n = 5); group 4, was fully kindled animals with at least 10 consecutive stage 5 seizures (n = 5) and group 5, was operated but not kindled sacrificed at the end of the experiments (n = 6). Three hours after the last stimulation animals were decapitated and blood from the levetiracetam treated animals was collected and stored at -80°C.

**Table 1:** Animal groups, kindling and seizure parameters from the final day of stimulation

Group	Treatment	Animal number	Seizure Severity	Afterdischarge duration (s)	Seizure duration (s)	Total no. of stimulations	[LEV]_plasma_ mg/L	Amplitude (μA)
		CS1	-	-	-	-	-	-
		CS2	-	-	-	-	-	-
Control	-	CS3	-	-	-	-	-	-
start		CS4	-	-	-	-	-	-
		CS5	-	-	-	-	-	-

		3V1	3	68	n.d.	6	-	150
		3V2	3	69	n.d.	6	-	100
		3V3	3	8	n.d.	6	-	350
	Vehicle	3V4	3	41	n.d.	18	-	150
		3V5	3	71	n.d.	20	-	150
		3V6	3	7	n.d.	22	-	250
1		3V7	3	75	n.d.	22	-	150
		
		3L1	0	16	n.d.	6	19.7	450
		3L2	3	12	n.d.	6	33.2	225
	Levetiracetam	3L3	0	14	n.d.	6	31	325
	40 mg/kg	3L4	1	12	n.d.	18	24	350
		3L5	0	10	n.d.	20	23.1	200
		3L6	1	11	n.d.	22	20.1	300
		3L7	5	42	20	22	23.6	375

		5V1	5	46	41	23	-	325
		5V2	5	127	48	31	-	175
	Vehicle	5V3	5	105	44	36	-	225
		5V4	5	23	18	42	-	400
2		5V5	5	122	96	40	-	300
		
		5L1	1	37	n.d.	23	23.3	375
	Levetiracetam	5L2	0	83	n.d.	31	24.3	125
	40 mg/kg	5L3	5	18	10	36	27	325
		5L4	3	19	n.d.	42	n.d.	275
		5L5	3	71	n.d.	40	18.9	250

		FV1	5	95	41	14	-	250
		FV2	5	98	42	14	-	375
	Vehicle	FV3	5	120	55	19	-	200
		FV4	5	132	43	22	-	150
3		FV5	5	78	59	25	-	350
		
		FL1	5	13	12	14	19.6	275
	Levetiracetam	FL2	1	16	n.d.	14	27.5	200
	40 mg/kg	FL3	5	12	14	19	39.7	500
		FL4	1	9	n.d.	22	22.6	150
		FL5	5	69	31	25	18.5	100

		CE1	-	-	-	-	-	-
		CE2	-	-	-	-	-	-
Control	-	CE3	-	-	-	-	-	-
end		CE4	-	-	-	-	-	-
		CE5	-	-	-	-	-	-
		CE6	-	-	-	-	-	-

n.d., not determined

### Gene expression assays

Following decapitation, hippocampi were quickly dissected and total RNA from the entire hippocampus (dorsal and ventral regions) was separately purified using the RNeasy Mini Kit (Qiagen, Hilden, Germany). Prior to the isolation procedure tissue was disrupted by a pestle fitting into an Eppendorf tube and lysed in lysis buffer supplied with the kit. The RNA was on column DNase-treated for 30 minutes with Rnase-free DNase Set (Qiagen) and finally eluted in 2 times 30 μl of DEPC-treated H_2_O.

#### Differential Display RT-PCR (DDRT-PCR)

For the DDRT-PCR analysis we used pooled total RNA from the contralateral hippocampus of vehicle treated animals in the 3 kindling groups and the 2 control groups: control start (n = 5), stage 3 vehicle treated (n = 7), stage 5 vehicle treated (n = 5), fully kindled vehicle treated (n = 5) & control end (n = 6). The DDRT-PCR analysis was performed using 288 different primer combinations as described previously [30].

#### Quantitative RT-PCR

To further profile the kindling-induced alterations in gene expression quantitative RT-PCR of pooled total RNA was performed. Gene-specific primers were designed according to the following guidelines (Additional file 1, Table S1): an amplicon size of 50-150 basepairs, GC content within the 20-80% range, no runs of identical nucleotides and a Tm between 58-60°C. We used the Taqman^® ^Reverse Transcription reagents (Applied Biosystems, Foster City, CA) to perform first strand synthesis on 1 μg total RNA according to the manufacturer's prescriptions with 2.5 μM random hexamers by incubating a 100 μl reaction 10 minutes at 25°C, 30 minutes at 48°C and finally 5 minutes at 95°C. After synthesis the cDNA was diluted twice with Rnase-free H_2_O and 3 μl was used in a 20 μl quantitative RT-PCR reaction containing 1 × SYBR Green^® ^Mix (Applied Biosystems) and 7.5 pmol of each primer. The samples were analyzed on the DNA Engine Opticon (MJ Research, Waltham, MA) with cycle conditions as follows: 2 minutes at 50°C; 10 minutes at 95°C; 40 cycles of 15 seconds at 95°C and 1 minute at 60°C. Quantitative PCR measurements was either analyzed by applying the 2^-ΔΔC^T or the Pfaffl method [31-34]. In short, the relative expression level of each cDNA was calculated by normalizing to the expression levels of β-actin or cyclophilin A (PPIA) in the sample, and set relative to the mean normalized expression levels of the control samples. GAPDH was used as a negative control. Each quantitative RT-PCR experiment has been done twice of which only one representative experiment is shown. Triplicate qPCR data is presented as mean ± SEM. It is indicated in the figure legends when quantitative analysis of RNA from individual animals rather than pooled RNA has been performed.

#### In situ hybridization

Radioactive *in situ *hybridization with ^33^P-riboprobes was done on 12 μm coronal cryosections from amygdala-kindled and control rat brain. The brains were cut in a cryostat and transferred onto Superfrost Plus slides. Subsequently, cryosections were fixed in 4% PFA for 5 minutes, washed in PBS for 2 minutes, incubated for 2 minutes in acetic anhydride/triethanolamine, washed in PBS for 2 minutes and dehydrated in increasing concentrations of EtOH (from 30%-99%).

The differentially regulated DDRT-PCR bands were amplified using the downstream T7-promoter harbouring poly-T primer and a designed gene-specific upstream primer with a T3-promoter overhang. The riboprobes were *in vitro *transcribed from either the T7-promoter (antisense) or T3-promoter (sense) in the PCR-amplified fragments using the Maxiscript *in vitro *transcription kit (Ambion, Austin, TX). Recovery of the *in vitro *transcribed fragments was performed on NucAway Spin columns (Ambion) according to the manufacturer's instructions. Each probe was denatured for 3 min at 80°C and after cooling 1-2 μl probe was transferred to 150 μl hybridization buffer containing 50% 2 × HYBE solution (SIGMA), 50% deionised formamide, 10% dextrane sulphate and 1 mg/ml tRNA. The hybridization mix was transferred to the processed cryosections, covered with a coverslip and incubated ON at 60°C. After ON hybridization coverslips were removed in 1× SSC/50% formamide, washed in 2 × 30 minutes at 42°C in 1 × SSC/50% formamide, RNase-treated for 30 minutes at 37°C, dehydrated in increasing concentrations of EtOH (30%-99%) each containing 300 mM ammonium acetate, washed twice in 99% EtOH and finally exposed for 3-4 weeks on Biomax film (Kodak).

### Plasma concentrations of levetiracetam

Rats were decapitated 3 hours after the last i.p. injection of levetiracetam and full blood was collected in sodium-heparin tubes and centrifuged (5000 rpm) for 10 minutes. Subsequently, plasma was collected and frozen at -80°C until analysis. By using validated HPLC methods, solvent extraction and UV detection the concentration of levetiracetam in plasma was determined in duplicate and results were only accepted if the plasma levels determined were within 10% of each other. In the case of animal 5L4 the levetiracetam plasma concentration could not be determined. Hence, RNA from rat 5L4 and its levetiracetam paired animal 5V4 were not included in the subsequent qPCR analysis.

## Results

### Kindling procedure

One week after implantation of the stimulation electrodes the kindled rats were divided in two halves (Table 1). Half of the animals received vehicle and the other half received 40 mg/kg levetiracetam i.p. 1 hour prior to the kindling stimulation. In addition, the kindled animals were also grouped in kindling pairs, with one vehicle (V) treated rat and one levetiracetam (L) treated rat in each kindling pair. The initial pairing of animals secured that each levetiracetam treated rat was paired with a vehicle treated control rat, and that the number of stimulations received by the levetiracetam treated rat was matched to that of its pair-housed vehicle treated control rat. A consequence of this is that not all levetiracetam treated rats in the "Stage 3" group actually will reach Stage 3 seizures, not all levetiracetam treated in the "Stage 5" will reach Stage 5 seizures and so forth. Prior to the experiments, 5 electrode implanted non-kindled control animals (CS1-5; see Table 1) were sacrificed. During the experiment kindling pairs were sacrificed at 3 different kindling stages thus giving 3 different kindling groups. In the stage 3 group, a total of 7 kindling pairs (3V1-7 and 3L1-7; see Table 1) were selected when the vehicle animals reached stage 3 seizures; in that group, 3 kindling pairs (3V1-3 and 3L1-3) were selected and sacrificed after 6 stimulations and another 4 kindling pairs (3V4-7 and 3L4-7) were sacrificed after 18-22 stimuli. In the stage 5 group, 5 kindling pairs (5V1-5 and 5L1-5) were sacrificed when the vehicle animals reached the first or the second stage 5 seizure. In the fully kindled group, consisting of 5 kindling pairs (FV1-5 and FL1-5), animal pairs were sacrificed when the vehicle animals reached the fully kindled stage (10 consecutive stage 5 seizures). Finally, in order to control for variation in age related gene expression, six operated but non-kindled animals (CE1-6) were sacrificed at the end of the kindling experiment.

### Amygdala kindling

The mean number of stimulations needed for a rat to reach a stage 3 seizure was 8.9 ± 2.2 stimulations (mean ± SEM, n = 17) whereas the mean number of stimulations needed to reach the first stage 5 seizure was 20.4 ± 2.3 stimulations (mean ± SEM, n = 10). The mean AD duration on the last day of kindling for stage 3, stage 5 and fully kindled rats were 48.4s ± 11.4s, 84.6s ± 21.09s and 104.6s ± 9.6s (mean ± SEM, n = 5-7), respectively.

### Effect of levetiracetam in amygdala-kindled rats

**Figure 1:**
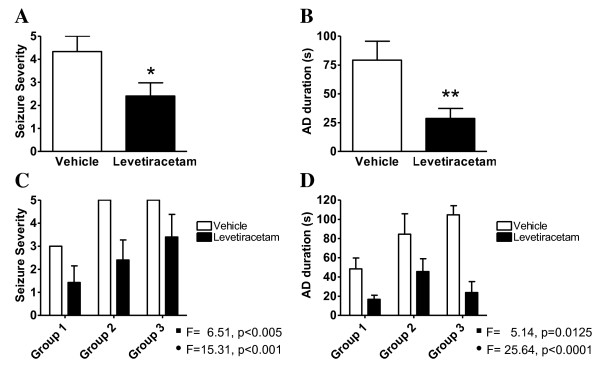
**Anti-epileptic effects of levetiracetam in the amygadala-kindled rat**. *A*. Seizure severity of amygdala-kindled rats on the final day of stimulation. *B*. After discharge duration of amygdala-kindled rats on the final day of stimulation. Animals in vehicle group (n = 17) and levetiracetam group (n = 17) were administred 0.9% NaCl solution or 40 mg/kg levetiracetam, respectively. Data is presented by mean values ± SEM. Asterisks indicate a significant difference in mean values between vehicle and levetiracetam groups. (p < 0.05, *; p < 0.01, **; Mann-Whitney test). *C*. Seizure severity of stage 3 rats (n = 7), stage 5 rats (n = 5) and fully kindled rats (n = 5) on the final day of stimulation. *D*. After discharge duration of stage 3 rats (n = 7), stage 5 rats (n = 5) and fully kindled rats (n = 5) on the final day of stimulation. 2-way ANOVA was performed with respect to kindling effect and levetiracetam effect. The F-statistics and p-values for the kindling effect (black square) and levetiracetam effect (black circle) in each ANOVA are indicated in the figure.

Levetiracetam significantly reduced the seizure severity (Figure 1A) and AD duration (Figure 1B) when compared to vehicle treated amygdala-kindled rats. Only 29% of the animals (5 of 17 animals) treated with levetiracetam experienced a stage 5 seizure compared to 59% of the vehicle treated animals. No linear correlation existed between the inability of levetiracetam to attenuate development of stage 5 kindling and the plasma levels of levetiracetam (see Table 1). The correlation coefficients for plasma levels versus seizure severity and AD duration were 0.13 (linear regression, r^2 ^= 0.1283, ns) and 0.36 (linear regression, r^2 ^= 0.017, ns), respectively.

A two-way ANOVA analysis showed that there was both a statistically significant kindling effect and a statistically significant levetiracetam effect in amygdala-kindled rats treated with levetiracetam when measured on parameters such as seizure severity (Figure 1C) and AD duration (Figure 1D). On the contrary there was no interaction between the drug and kindling suggesting that the effect of levetiracetam has no differential effect with respect to the different kindling groups. Administration of levetiracetam also lowered the seizure duration (17.4s ± 3.8s) significantly compared to vehicle treated amygdala-kindled rats (48.7s ± 6.3s, students t-test; P < 0.005). Four animals receiving levetiracetam showed no seizures and five animals developed seizures less severe than stage 3 (see Table 1 for last day stimulation parameters). Additionally, no animal receiving levetiracetam experienced AD durations of more than 90 s compared to 7 vehicle animals. No behavioural adverse effects of the drug treatment were observed during the entire kindling experiment.

### Identification of differentially expressed mRNAs in vehicle treated amygdala-kindled rats

**Figure 2:**
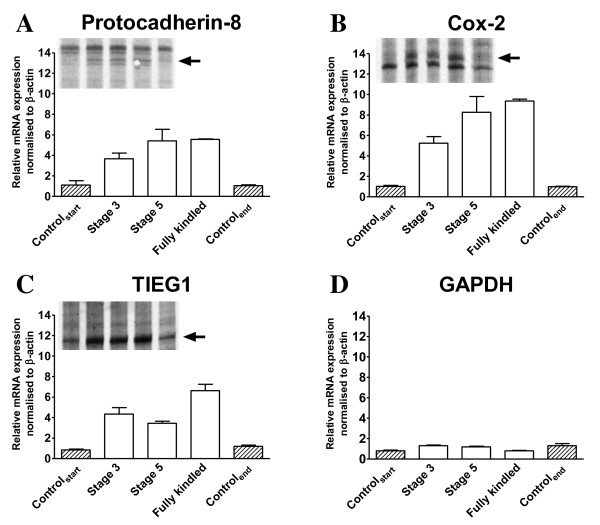
**Identification and verification of differential expressed Pcdh-8, TIEG1 and COX-2 transcripts in the ipsilateral hippocampus of amygdala-kindled rats 3 hrs after the last seizure**. Quantitative RT-PCR on total RNA from control and amygdala-kindled rats of *A*. Pcdh-8, *B*. COX-2, *C*. TIEG1 and *D*. GAPDH. The relative expression level of each cDNA was calculated by the ΔΔ-method, normalized to the expression levels of β-actin in the sample, and set relative to the mean normalized expression levels of the control samples. GAPDH was used as a control. Each quantitative RT-PCR experiment has been done twice of which one representative experiment is shown. Triplicate qPCR data is presented as mean ± SEM. *Top inserts A-C*, autoradiograms showing the DDRT-PCR bands corresponding to Pcdh-8, COX-2 and TIEG1, respectively. *Arrow *indicates the DDRT-PCR analysed bands. The 5 lanes correspond to control start, stage 3, stage 5, fully kindled and control end, respectively.

**Figure 3:**
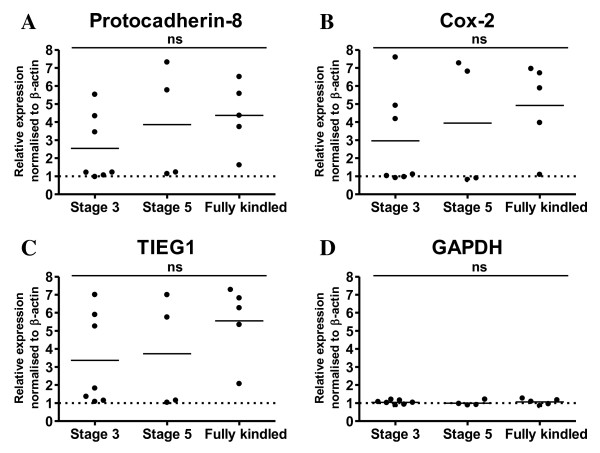
**Quantitative RT-PCR on total RNA from the contralateral hippocampus of individual control and amygdala-kindled rats of *A*. Pcdh-8, *B*. COX-2, *C*. TIEG1 and *D*. GAPDH**. The relative expression level of each cDNA was calculated by the ΔΔ-method, normalized to the expression levels of β-actin in the sample. GAPDH was used as a control. The qPCR data is presented as mean ± SEM.

To identify differentially expressed transcripts during kindling acquisition, total RNA was purified from the ipsilateral hippocampus of 2 groups of non-kindled and 3 groups of vehicle treated kindled rats 3 hours after their last seizure activity and subsequently, pooled and screened for transcriptional differences by the Differential Display RT-PCR (DDRT-PCR) method [35]. In all 3 kindling stages compared to the non-kindled controls Cox-2, Pcdh-8 and TIEG1 were identified as differentially expressed transcripts (Figure 2A-C, *top inserts*). No change in GAPDH levels were observed (Figure 2D). Quantitative RT-PCR with primers amplifying Cox-2, TIEG1 and Pcdh-8 revealed a good correlation between the ipsi- and contralateral hippocampus in the relative expression of kindling-induced gene expression (Additional File 1, Figure S1). This suggests that expression levels in the contralateral hemisphere are predictive for the expression levels in the ipsilateral hemisphere at the analyzed kindling stages (≥ stage 3). On the contrary, quantitative RT-PCR analysis done on total hippocampal RNA from individual rats shows that there was no significant difference in the expression levels between the individual kindling stages of Cox-2, Pcdh-8 and TIEG1 (Figure 3A-D) suggesting either that a) the induction of these genes in the hippocampus are not underlying the epileptogenic process or b) that the epileptogenic process is ongoing at earlier time-points. Localization of the kindling-induced transcripts of Pcdh8 and TIEG1 was determined to the dentate gyrus by *in situ *hybridization (ISH) on cryosections from stage 3 amygdala kindled and control rats with ^33^P labelled riboprobes (Additional file 1, Figure S2) which fits with previous observations for Cox-2 [36,37].

### Differential expression of "synaptic plasticity related IEGs" in amygdala-kindled rats

Based on the differential expressed transcripts identified in the DDRT-PCR analysis and expression changes of genes related to those transcripts (data not shown) we examined the literature for genes previously reported as involved in models of neuronal or synaptic activity including maximal electroconvulsive shock, kainate induced seizures, long-term potentiation, long-term depression, chronic epilepsy models including amygdala-hippocampal kindling as well as models with spontaneous seizure activity after pilocarpine- and PTZ- induced status epilepticus. We identified 105 genes from these studies (see Additional file 1, Table S1 for genes and primer sequences) and then examined the mRNA expression levels by quantitative PCR of total RNA isolated from the ipsilateral hippocampus of control and amygdala-kindled rats at different kindling stages (see Additional file 1, Table S2 for relative mRNA levels). Nineteen genes including the 3 genes identified by DDRT-PCR were verified as differentially expressed in the amygdala-kindled rat relative to non-kindled control rats (Additional file 1, Table S3 and Table S4). Some genes that previously have been strongly linked to amygdala-kindling such as NPY [38] only showed nominal differential expression compared to either of the control groups and therefore were omitted from further analysis. Many of the 19 qpcr validated transcripts have previously been identified as IEGs and fewer genes such as synaptopodin and beta-A activin as late response genes (for review see Lanahan, 1998) [39]. The levels of cyclophilin A and GAPDH, which were included as controls showed no differential expression in amygdala-kindled rats compared to control animals.

### Effects of levetiracetam on kindling-induced gene expression

**Figure 4:**
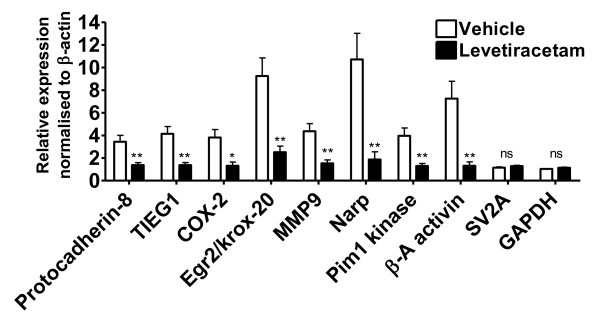
**Gene expression profiling the effects of levetiracetam on kindling-induced genes**. Quantitative RT-PCR of RNA from the contralateral hippocampus of levetiracetam and vehicle treated amygdala-kindled rats with primers against 8 seizure-induced genes and GAPDH and SV2A as controls. All data are expressed relative to animal FL3, normalized to β-actin levels and expressed as mean ± SEM (n = 16). Asterisks indicate a statistical significant difference in mean values between vehicle and levetiracetam groups. (p < 0.05, *; p < 0.01, **; Paired t-test).

The effect of the anti-epileptic drug levetiracetam on the identified kindling-induced genes was determined using quantitative RT-PCR on total ipsilateral hippocampal RNA from control, vehicle and levetiracetam animals and on total contralateral hippocampal RNA from vehicle and levetiracetam animals (Table 2 and Additional file 1, Figure S3). The housekeeping gene GAPDH was included as control and all expression levels were normalized to the β-actin levels. A subset of genes was also tested on the single animal level and here levetiracetam when compared to vehicle significantly attenuated the kindling-induced expression in the hippocampus of amygdala-kindled rats in all of the analyzed transcripts (Figure 4). There was no significant difference in the mRNA levels of the control transcripts GAPDH and the levetiracetam target SV2A when comparing levetiracetam animals to vehicle controls. Also to rule out a general detrimental effect of levetiracetam on hippocampal neurons the mRNA expression levels of a set of constitutively expressed neuronal mRNAs were measured. There was no observed significant effect of levetiracetam compared to vehicle on the mRNA levels of neuronal transcripts such as Homer-1b/c, CAMKII, GluR2, Synapsin I and Synaptophysin (Additional file 1, Figure S4 and Table S3).

**Table 2:** Effects of levetiracetam on kindling-induced IEG expression in the hippocampi of kindled rats

	Ipsilateral				Contralateral
		
Functional group	mRNA	Vehicle†	Levetiracetam†	% reduction	% reduction
	Egr3/Pilot	6.9 ± 0.4	2.4 ± 0.2	66	69
Transcription factors	c-fos	5.8 ± 0.7	1.3 ± 0.1	77	67
	Egr2/krox-20	12.5 ± 0.7	3.1 ± 0.2	75	88
					
	NGF	1.4 ± 0.1	0.8 ± 0	45	9
Neurotrophic factors	BDNF	7.4 ± 0.8	2.4 ± 0.1	67	70
	NT-3	0.7 ± 0	0.9 ± 0.1	-22	-31
					
TGF-β	β-A activin	13.1 ± 1.1	3 ± 0.1	77	89
superfamily	TIEG1	6.2 ± 0.5	1.5 ± 0.1	76	72
					
Inflammatory	TNF-α	4 ± 0.3	1.1 ± 0.1	74	54
response	Cox-2	7.6 ± 0.5	2 ± 0.1	74	83
					
	Homer-1a	10.6 ± 1	2.9 ± 0.2	73	77
GPCR interactions	Ania-3	3.2 ± 0.2	2.5 ± 0.1	21	32
	RGS-2	3.2 ± 0.1	0.8 ± 0.1	73	61
					
Extracellular	MMP-9	6.7 ± 0.3	2.2 ± 0.2	67	59
proteins	Narp	20.5 ± 1.8	2.5 ± 0.1	88	90
					
Cell adhesion	Protocadherin-8	5.1 ± 0.3	1.1 ± 0.1	78	80
proteins	Arc	15.9 ± 1.7	2.9 ± 0.1	82	78
					
	Synaptopodin	2.1 ± 0.2	1.4 ± 0.1	33	38
Others	Pim-1 kinase	4.3 ± 0.2	1.3 ± 0	70	71
	GAPDH	1.2 ± 0.1	0.9 ± 0	22	14

^† ^relative expression ± SEM to control start normalized to β-actin

**Figure 5:**
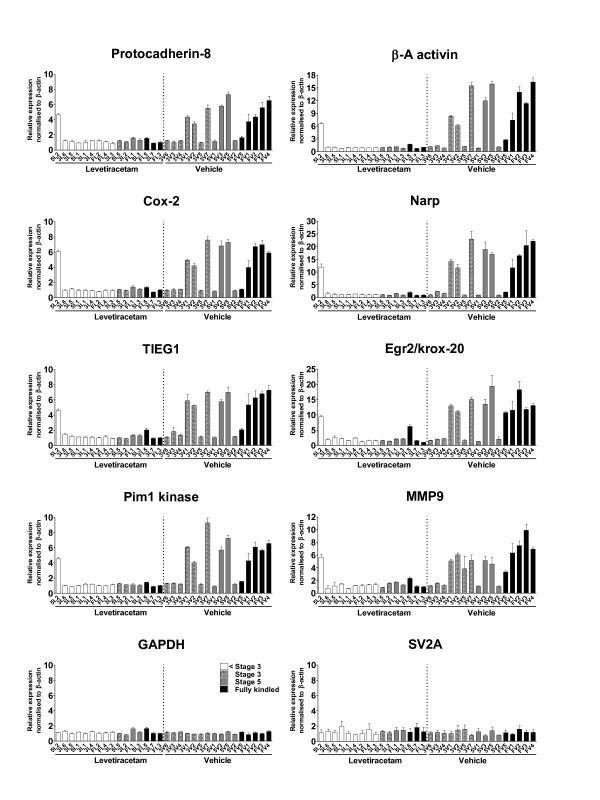
**Single animal profiling the effects of levetiracetam on kindling-induced genes in amygdala-kindled rats**. Quantitative RT-PCR of RNA from the contralateral hippocampus of levetiracetam and vehicle treated amygdala-kindled rats with primers against 8 seizure-induced genes and GAPDH and SV2A as control. Vehicle treated rats and levetiracetam treated animal pairs are numbered according to Table 1. In the vehicle and the levetiracetam groups shaded, grey and black bars represent stage 3, stage 5 and fully kindled animals, respectively. White bars represent lower than stage 3. All data are expressed relative to animal FL3, normalized to β-actin levels and expressed as mean ± SEM.

The overall efficacy of levetiracetam to attenuate the kindling-induced IEG expression in both the ipsi- and contralateral hippocampus was determined. Here, it was found that the average reduction in kindling-induced expression by levetiracetam was approximately 65% in both the ipsi- and the contralateral hippocampus (Table 2). A linear correlation was observed between the percentage reductions by levetiracetam in the ipsi- versus the contra-lateral hippocampus (correlation coefficient 0.93; linear regression, r^2 ^= 0.8622, P < 0.001). Next, we wanted to examine the effect of levetiracetam on inter-individual gene expression. However, when plotted individually a large underlying inter-individual variation in the kindling-induced gene expression was observed in the vehicle animals while the inter-individual variation, as well as the absolute expression changes, was markedly lower in the levetiracetam group indicating that chronic application of 40 mg/kg levetiracetam had a potent attenuating effect on the kindling-induced IEG expression (Figure 5). Only one animal showed significant differences in gene expression levels compared to the rest of the levetiracetam-treated group; nevertheless, animals expressing all kindling stages are represented in the levetiracetam group.

In contrast, 80% of the fully kindled rats in the vehicle group showed enhanced expression levels for all tested genes whereas 50% of the stage 5 animals and only 43% of the stage 3 animals exhibited enhanced expression levels of the 8 examined genes. No inter-individual differences in the relative expression levels of SV2A and GAPDH were observed.

### Rats with kindling-induced high IEG expression have longer AD duration

**Figure 6:**
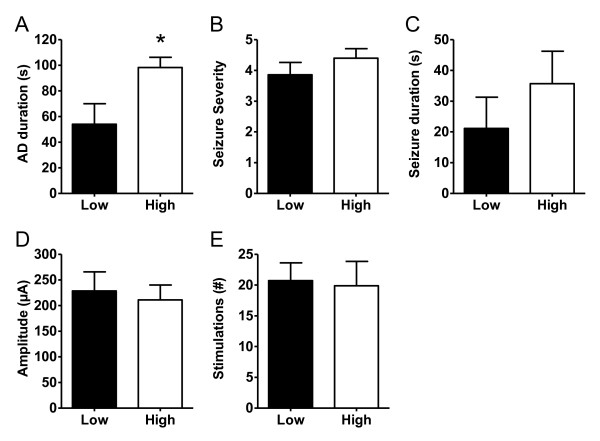
**Comparison of "High IEG" expressing (n = 9) and "Low IEG" expressing (n = 7) amygdala-kindled vehicle treated rats with respect to *A*. AD duration, *B*. seizure severity, *C*. seizure duration, *D*. stimulation amplitude, and *E*. stimulation number**. "High IEG" were 3V1, 3V7, 3V2, 5V3, 5V5, FV1, FV2, FV4 and FV3 whereas "Low IEG" animals were 5V2, FV5, 3V6, 3V5, 5V1, 3V4 and 3V3.All data are expressed as mean ± SEM. Asterisks indicate a statistical significant difference in mean values between vehicle and levetiracetam groups. (p < 0.05, *; Unpaired t-test and Mann-Whitney test).

In individual animals, we correlated the observed changes in gene expression with kindling parameters such as AD duration, seizure duration, seizure severity, and stimulation number and stimulation amplitude. However, no correlations at the single animal level were found (data not shown). Based on the data presented in Figure 5, which suggests that there are two discrete populations of IEG responders, a one-way ANOVA was performed on each of the individual gene data. The statistical analysis for 6 of the individual 8 genes consistently identified 7 vehicle treated kindled animals as having no significant differences in individual gene expression compared to all the levetiracetam treated kindled animals (except the outlier 5L2) (Tukey's Multiple Comparison Test, p > 0.05, n = 3). Vice versa, 9 vehicle treated kindled animals did have significant differences in individual gene expression compared to all levetiracetam animals (except the outlier 5L2) (Tukey's Multiple Comparison Test, p < 0.05, n = 3). These animals were categorized as "low IEG" and "high IEG" expressing animals, respectively (see Figure 6 legend for animal identifiers). Based on that analysis, we hypothesized that kindling-induced gene expression in the hippocampus is an "all-or-none response" that depends on the progression of seizure activity from the amygdala to the hippocampus. Indeed, "high IEG" expressing kindled rats have an AD duration that is almost twice as long as "low IEG" expressing kindled rats indicating that longer AD duration times increase the likelihood of hippocampal involvement in the seizure thereby giving rise to increased gene expression (Figure 6). In support, the only levetiracetam treated amygdala-kindled rat (5L2), which exhibited "high IEG expression" also had a very long AD duration time of 83 s. With respect to seizure severity, seizure duration, stimulation number and stimulation amplitude no statistically significant differences were found between "high IEG" and "low IEG" kindled rats (Figure 6).

## Discussion

This study has 3 main findings. 1) Cox-2, TIEG1 and Pcdh-8 are differentially expressed in the hippocampus during the epileptic process in the kindled rat and the differential expression is dependent on the duration of the AD measured by the recording electrode placed in the amygdala. 2) All 3 genes belong to a seizure-induced IEG expression profile that also comprises at least 16 other genes. Many of these genes have previously been described in various models of synaptic plasticity. 3) In our study, we also found that the anti-epileptic drug levetiracetam attenuated the kindling-induced changes of both immediate early and late response gene expression 3 hours after the last seizure. Previously, the effect of levetiracetam on kindling-induced gene expression after 1 hour has been reported for two single genes BDNF and NPY [40] and after 24 hours for NPY, TRH and GFAP [41] but never after 3 hours on a larger panel of both IEG and late response genes. Consequently, our data further expands the list of transcripts sensitive to the actions of levetiracetam. In addition, we also found that the attenuating effect of levetiracetam on seizure-induced gene expression was paralleled by the drug effect on AD duration which has not been reported previously. These findings suggest that levetiracetam during epileptic seizures targets mechanisms that reduce the AD duration which in turn prevents the increase in mRNA expression of synaptic plasticity-related genes in areas undergoing synaptic remodelling in response to the enhanced neuronal activity which is reported to occur both *in vitro *and *in vivo *[42,43].

### Kindling-induced expression of a subset of genes associated with synaptic remodeling

Utilising a DDRT-PCR based approach to find gene expression changes in the hippocampus during amygdala-kindling Cox-2 was identified as differentially expressed. Cox-2 has previously been associated with epileptic conditions in both animals and humans [44,45]. Two additional kindling-induced transcripts were identified and validated by the DDRT-PCR method. Of these, TIEG1 was originally identified as differentially expressed in human osteoblasts after TGF-β activation [46]. It is induced in oligodendroglial precursor cells after TGF-β treatment [47] and in rat brain after kainate-induced seizures [48]. TGF-β is the most potent inducer of TIEG1 but other members of the TGF-β superfamily also induce TIEG1 including GDNF, BMP-2 and β-A activin [49,50]. In contrast, information on signaling cascades involving the cell-adhesion molecule Pcdh-8 is very limited. Pcdh-8 is predominantly expressed in the nervous system and during development [51] and it has previously been implicated in neuronal plasticity, long-term potentiation and synaptic remodelling [52,53].

Subsequently, a bioinformatics approach combined with quantitative RT-PCR lead to the identification of 16 additional kindling-induced genes. The differential expressed transcripts fall into the following functional categories: transcription factors (c-fos, krox-20 and egr3), neurotrophic factors (NGF, BDNF and NT-3), inflammatory response (Cox-2 and TNF-α), extracellular matrix components (Narp and MMP9), cell-adhesion (Pcdh-8), TGF-β superfamily signaling (TIEG1 and β-A activin), GPCR signaling (RGS2, Homer1a and Ania3) and others (Pim1 kinase, Arc and Synaptopodin). Historically, many of these transcripts have been linked to synaptic plasticity and models of enhanced neuronal activity [39,54,52,73]. In agreement, DDRT-PCR studies in rapidly kindled mice revealed differential expression of 26 transcripts amongst those RGS2, krox-24, Homer and c-fos [74] and differential expression of Cox-2, TIEG1, Narp, Arc, Ania-3, Homer1a/Vesl and BDNF are also seen in the hippocampus after electroconvulsive shock [75]. Two of the differentially expressed transcripts Homer-1a and Ania-3 originate from the same Homer 1 genetic loci [57]. However, we rule out a more general transcriptional activation of the Homer 1 gene in amygdala-kindled rats as a third splice variant of the Homer1 gene Homer-1b/c is not differentially expressed after kindling-induced seizures (see Additional file 1, Table S2). Consequently, we propose that the kindling-induced transcripts are molecular indicators of immediate early changes, which are associated with enhanced neuronal activity in the hippocampus in models of synaptic plasticity including epilepsy models. Whether any, just a subset or all of these genes are causal for the kindling process still remains to be verified.

### Induction of IEGs in the hippocampus seems to be an "all-or-none" effect

The expression of c-fos mRNA has previously been shown induced ipsilaterally after only a few ADs. However, upon repeated stimulations the kindling-induced c-fos mRNA expression progresses to the contralateral hippocampus in animals at stage 3 or more; at later stages kindled animals exhibit no asymmetry in c-fos expression [76]. Likewise, we also found a good correlation between the ipsi- and contralateral hippocampus in the normalized expression levels of Pcdh-8 and Cox-2 in stage 3 and stage 5 kindled rats. The progression of mRNA expression of these genes from ipsilateral to contralateral regions during progression of kindling at earlier stages than stage 3 also indicates that these genes are induced as a consequence of the spread of seizure activity from ipsilateral to contralateral regions. Overall, we observed no significant difference in the IEG mRNA levels between stage 3, stage 5 and fully kindled populations. However, when analysing individual animals, a higher frequency of IEG mRNA induction was seen at the later stages compared to earlier kindling stages. This could indicate an "all-or-none" effect in transcriptional induction rather than a gradual increase in the transcriptional response when animals progress from stage 3 to fully kindled. At the single animal level kindling-induced IEG expression was observed in half of the stage 5 and 80% of the fully kindled rats, respectively. So even though the incident rate of IEG induction increases with kindling acquisition not all kindled animals have changes in hippocampal IEG expression, which is also in good agreement with previous studies [76,77]. In addition, at the single animal level there seems not to be a linear correlation between seizure parameters like seizure severity and AD duration and the relative mRNA expression levels of the examined genes (Figure 5) suggesting an "all or none" effect of kindling on IEG induction in the hippocampus. In support, kindled rats with "high IEG" expression also have significant longer AD duration times than kindled rats with "low IEG" expression.

### Levetiracetam reduces the probability for induction of IEG transcripts involved in synaptic remodeling in the hippocampus

In line with other studies we show that levetiracetam exhibits an anti-epileptic effect in the amygdala-kindling model, i.e. animals pre-treated with levetiracetam have shorter AD duration and lower seizure severity compared to non-treated controls [11,12]. In our study, the anti-epileptic effect paralleled the observation that levetiracetam also exerted a potent inhibitory effect on kindling-induced changes in IEG mRNA expression in the hippocampus. However, analysis of the individual animals revealed that only one of the 16 levetiracetam treated animals showed a significant change in the IEG mRNA levels, whereas 9 of the 16 vehicle treated animals exhibited robust changes in IEG mRNA levels. Nevertheless, 5 of 16 levetiracetam treated animals developed stage 5 seizures during the treatment. No difference in the mRNA levels of the levetiracetam target SV2A as well as in a number of other neuronal genes was observed hence ruling out compensatory mechanisms as sensitization/desensitization of SV2A or even a more general toxicity effect of the drug on neuronal expressed mRNAs. Conclusively, this suggests that levetiracetam might exert part of its anti-epileptic effect by reducing the likelihood of an "all-or-none" induction of genes putatively involved in establishing long term synaptic changes important to kindling acquisition, synaptic plasticity and perhaps epileptogenesis.

Expression of the IEG expression is not necessarily induced during neuronal hypersynchronous seizure activity in the hippocampus as it also requires bursts of population spikes [77]. This has been further supported by human studies showing correlation between spiking activity and gene expression changes [78]. Levetiracetam is able to inhibit hypersynchronous activity by reducing the epileptiform activity-induced population spikes in the CA3 area of the hippocampus [79-82]. This suggests that levetiracetam is attenuating the kindling-induced IEG mRNA expression by targeting mechanisms that reduces the epileptiform activity-induced population spikes in the hippocampus. However, it would require a recording electrode in the hippocampus to be able to correlate the AD duration time, existence of population spike activity in the hippocampus and increased IEG expression to the attenuating effects of levetiracetam.

## Conclusions

In conclusion, our data suggest that levetiracetam is a potent inhibitor of the immediate early transcriptional responses to episodes of enhanced neuronal spiking activity in the hippocampus. Given that many of these transcripts are encoding proteins involved in synaptic remodeling also suggests that levetiracetam could inhibit structural changes in areas undergoing synaptic plasticity.

## Abbreviations

AD: afterdischarge; Cox-2: Cyclooxygenase-2; IEG: immediate early gene; Pcdh8: Protocadherin-8; DDRT-PCR: Differential Display RT-PCR; qPCR: quantitative PCR; TIEG1: TGF-beta-inducible early response gene-1.

## Authors' contributions

KVC did all the experimental work except from the HPLC measurements, participated in the data analysis/data interpretation and drafted the manuscript. HL participated in the design, planning and the subsequent data analysis of the DDRT-PCR experiments. WPW participated in the interpretation of the amygdala-kindling data and HPLC measurements. CS participated in the design and planning of the amygdala-kindling as well as the data interpretation. PK participated in the design of the PCR and ISH experiments and manuscript revision. JE participated in designing and planning all experiments. JE also participated in the data interpretation and manuscript revision. All authors read and approved the final manuscript.

## Supplementary Material

Additional file 1 **Supplemental tables and figures**. Table S1: Primers and corresponding sequences used for qRT-PCR. Table S2: Initial qRT-PCR results. Table S3: Primers and corresponding sequences for validated genes and controls. Table S4: Comparison of ipsi- and contralateral expression levels of transcripts. Figure S1: In situ hybridisation autoradiograms of Pcdh8 and TIEG1. Figure S2: List of validated kindling-induced IEGs. Figure S3: Effect of LEV on kindling-induced Pcdh8, TIEG and Cox-2 expression. Figure S4: No effect of LEV on synaptic expressed control genes. Click here for file
