# Supplementary material for: Levetiracetam attenuates hippocampal expression of synaptic plasticity-related immediate early and late response genes in amygdala-kindled rats
Source: BMC Neurosci. 2010 Jan 27;11:9. doi: 10.1186/1471-2202-11-9 (PMC2848232; doi:10.1186/1471-2202-11-9)
Supplement: Additional file 1 — Supplemental tables and figures. Table S1: Primers and corresponding sequences used for qRT-PCR. Table S2: Initial qRT-PCR results. Table S3: Primers and corresponding sequences for validated genes and controls. Table S4: Comparison of ipsi- and contralateral expression levels of transcripts. Figure S1: In situ hybridisation autoradiograms of Pcdh8 and TIEG1. Figure S2: List of validated kindling-induced IEGs. Figure S3: Effect of LEV on kindling-induced Pcdh8, TIEG and Cox-2 expression. Figure S4: No effect of LEV on synaptic expressed control genes. [file 1471-2202-11-9-S1.PDF]

Table S1

Table S1. List of initial QPCR results

Gene name/Identifier/Acc #	Ctrl start		Ctrl end		Stage 3		Stage 5		Fully kindled	
	Mean	*St.dev*	*Mean*	*St.dev*	*Mean*	*St.dev*	*Mean*	*St.dev*	*Mean*	*St.dev*
ADT2	1.00	0.16	1.42	0.26	1.44	0.11	1.25	0.10	1.27	0.17
Alpha-spectrin 2	1.00	0.15	0.91	0.07	1.00	0.06	0.94	0.13	0.93	0.20
Ania3	1.00	0.25	0.94	0.18	2.48	0.24	1.86	0.32	3.49	0.78
APPBP2	1.00	0.15	0.83	0.11	0.83	0.02	0.85	0.06	1.10	0.10
Arc	1.00	0.27	1.22	0.25	7.30	2.86	7.04	5.48	13.48	3.08
Arcadlin	1.00	0.31	0.82	0.11	2.33	0.17	2.89	0.61	3.89	0.66
ATPsynthase6	1.00	0.41	0.53	0.05	0.72	0.04	0.62	0.15	1.13	0.62
bad2	1.00	0.46	1.42	0.19	0.73	0.24	0.96	0.24	1.01	0.21
bdnf	1.00	0.29	1.63	0.59	3.58	0.05	7.06	2.38	11.99	5.78
beta_actin	1.00	0.02	0.90	0.18	0.64	0.06	0.65	0.08	0.62	0.27
BI303981	1.00	0.43	1.11	0.10	0.50	0.08	0.91	0.16	0.62	0.07
c-fos	1.00	0.32	1.33	0.53	6.17	1.15	14.36	1.35	13.23	2.82
C/EBPb	1.00	0.14	0.84	0.12	0.70	0.09	0.66	0.16	1.26	0.28
Cdk5	1.00	0.43	0.69	0.05	0.73	0.16	0.66	0.06	0.85	0.10
CGI-12	1.00	0.81	0.73	0.32	0.50	0.34	0.85	0.23	1.35	1.59
cGolgip28	1.00	0.04	1.16	0.34	0.99	0.29	0.74	0.25	1.50	0.76
CO I	1.00	0.06	1.10	0.11	0.85	0.07	1.06	0.03	0.82	0.08
CO II	1.00	0.06	1.17	0.40	0.46	0.04	0.82	0.13	0.77	0.28
COX2	1.00	0.12	0.89	0.13	2.26	0.16	4.90	1.28	6.57	2.68
CRBP2C2KVC60	1.00	0.24	1.32	0.50	1.16	0.19	0.85	0.11	0.73	0.18
CyB	1.00	0.13	0.73	0.07	0.68	0.11	0.92	0.24	0.66	0.08
cyclophilin	1.00	0.30	0.95	0.34	0.63	0.09	0.75	0.07	1.16	0.14
CystatinC	1.00	0.24	1.82	0.57	1.04	0.67	0.98	0.37	0.83	0.27
DAPIT	1.00	0.23	1.06	0.16	0.80	0.11	1.06	0.30	1.25	0.78
DDX1	1.00	0.55	0.35	0.04	0.43	0.03	0.44	0.06	1.64	0.66
Dendrin	1.00	0.16	0.79	0.04	0.91	0.15	0.74	0.15	1.02	0.32
DRP2	1.00	0.29	0.99	0.20	0.61	0.13	1.25	0.26	0.79	0.43
Egr3	1.00	0.37	0.99	0.08	2.49	0.49	3.42	0.85	3.94	0.69
EST00098	1.00	0.46	0.64	0.12	0.77	0.52	0.51	0.12	0.69	0.15
FGF-2	1.00	0.09	1.01	0.27	0.91	0.27	0.82	0.24	1.17	0.16
GABAT	1.00	0.19	0.99	0.19	0.82	0.18	0.94	0.29	1.03	0.29
GAPDH	1.00	0.11	1.14	0.03	0.89	0.20	1.07	0.18	1.29	0.15
GCbGT	1.00	0.13	0.96	0.09	0.83	0.13	0.88	0.15	0.90	0.07
GDNF	1.00	0.15	1.28	0.28	1.08	0.25	1.49	0.96	2.21	0.99
Glypican4	1.00	0.38	1.00	0.04	0.89	0.57	1.10	0.17	1.18	0.14
GST3-DD4G1KVC38	1.00	0.24	0.73	0.05	0.80	0.09	0.80	0.09	1.05	0.21
H-ATPase	1.00	0.34	0.84	0.12	0.67	0.16	1.70	0.34	1.62	0.65
homer1a	1.00	0.07	1.12	0.17	6.64	1.14	6.74	1.04	10.09	1.32
homer1b/c	1.00	0.24	0.95	0.13	1.09	0.13	1.25	0.13	1.32	0.46
HSP70-B1A1KVC49	1.00	0.04	0.83	0.12	0.56	0.08	0.88	0.04	0.94	0.11
IL-1alpha	1.00	0.51	1.16	0.21	1.11	0.01	1.54	0.31	1.19	0.19
IL-6	1.00	0.13	0.73	0.09	0.83	0.08	0.92	0.09	0.92	0.11
KIAA0833	1.00	0.22	1.17	0.24	1.07	0.04	1.28	0.39	1.14	0.17
KID1	1.00	0.08	0.90	0.01	1.31	0.18	1.17	0.19	1.52	0.55
Kif21a	1.00	0.25	0.82	0.11	0.44	0.13	0.61	0.09	0.83	0.09
krox20	1.00	0.13	2.07	0.16	3.93	1.29	5.61	0.81	5.89	0.86
krox24	1.00	0.17	1.00	0.09	1.01	0.14	1.43	0.07	1.19	0.41
TIEG1	1.00	0.17	1.69	0.22	2.08	0.08	2.69	0.08	1.46	0.30
mGluR4	1.00	0.79	0.60	0.09	0.76	0.31	0.74	0.06	1.30	0.88
mGluR7	1.00	0.05	0.74	0.11	0.76	0.09	0.88	0.28	0.77	0.13
MIF2	1.00	0.18	0.63	0.08	0.27	0.06	0.36	0.11	0.78	0.29
mit.F1-ATPase	1.00	0.10	1.09	0.05	0.93	0.03	1.14	0.30	0.91	0.07
mLIM13C1KVC56	1.00	1.32	0.22	0.06	0.13	0.07	0.23	0.20	0.46	0.36
mLIM18G1KVC32	1.00	0.10	1.03	0.12	0.76	0.08	0.96	0.02	0.77	0.08
MAP1B	1.00	0.20	1.16	0.22	0.69	0.15	0.65	0.15	0.79	0.06
MMP-2	1.00	0.11	0.97	0.26	1.39	0.71	1.03	0.30	1.65	0.79
MMP-9	1.00	0.49	0.79	0.32	2.54	0.61	4.10	0.69	5.92	2.12
MSS4	1.00	0.40	0.67	0.11	0.28	0.07	0.75	0.13	0.83	0.65
mtGPDH	1.00	0.30	0.84	0.19	0.70	0.12	0.73	0.07	1.13	0.57
mVIMC2KVC54	1.00	0.19	1.12	0.09	0.96	0.11	1.05	0.16	0.70	0.02

Table S1(continued)

Table S1. List of initial QPCR results (continued)

	Ctrl start		*Ctrl end*		*Stage 3*		*Stage 5*		*Fully kindled*	
**Gene name/Identifier/Acc #**	***Mean***	***St.dev***	***Mean***	***St.dev***	***Mean***	***St.dev***	***Mean***	***St.dev***	***Mean***	***St.dev***
MyoVa	1.00	0.09	0.99	0.10	0.95	0.10	1.07	0.13	1.14	0.34
Narp	1.00	0.16	0.92	0.16	8.35	3.06	14.44	0.97	32.66	18.35
NCS1	1.00	0.37	0.76	0.24	1.10	0.30	1.22	0.14	0.88	0.27
Neuregulin	1.00	0.31	0.68	0.04	0.80	0.19	0.79	0.10	0.96	0.30
Neuritin	1.00	0.45	0.73	0.13	1.16	0.22	1.22	0.21	1.52	0.34
NGF	1.00	0.11	0.98	0.20	0.75	0.08	1.61	0.11	3.07	0.84
NPY	1.00	0.20	1.06	0.37	0.99	0.24	1.50	0.30	1.88	0.68
NSF	1.00	0.01	0.83	0.32	0.94	0.21	1.40	0.74	0.86	0.21
NT-3	1.00	0.16	0.84	0.07	0.56	0.11	0.54	0.13	0.63	0.22
NT-4	1.00	0.15	0.98	0.38	1.03	0.31	1.13	0.33	1.07	0.32
PCPTP1	1.00	0.11	0.69	0.07	0.50	0.22	0.50	0.17	0.65	0.18
PCTAIRE2	1.00	0.07	1.27	0.12	0.94	0.04	1.41	0.33	1.18	0.12
Pim1	1.00	0.53	1.15	0.33	3.04	0.20	3.64	1.06	7.09	2.27
RACK1	1.00	0.20	1.04	0.06	1.74	1.09	1.63	0.48	2.84	1.99
rb-A activin	1.00	0.63	0.83	0.07	6.75	1.25	13.04	0.65	13.81	1.66
RB3	1.00	0.46	1.09	0.19	0.71	0.18	1.64	0.54	1.24	0.74
RGS2	1.00	0.42	0.83	0.05	1.51	0.44	2.35	0.61	2.93	1.55
Rheb	1.00	0.22	0.93	0.09	1.07	0.29	1.13	0.16	1.75	0.20
rKCNQ1	1.00	0.09	4.86	0.34	0.41	0.19	3.43	0.24	2.49	0.71
rKCNQ2	1.00	0.28	0.59	0.07	0.73	0.10	0.64	0.20	0.78	0.31
rKCNQ3	1.00	0.62	1.04	0.39	0.70	0.22	1.24	0.44	0.99	0.91
rKCNQ4	1.00	0.43	0.90	0.19	0.69	0.23	0.85	0.25	1.42	1.07
rKCNQ5	1.00	1.42	0.25	0.08	0.15	0.02	0.40	0.36	0.14	0.04
RPL13	1.00	0.06	1.00	0.15	0.99	0.14	1.07	0.11	1.15	0.08
RPL19	1.00	0.05	1.64	0.27	1.25	0.46	1.35	0.18	1.20	0.15
RPL35	1.00	0.06	1.09	0.18	0.86	0.09	0.98	0.08	0.76	0.17
RPL41	1.00	0.07	1.03	0.09	0.93	0.05	0.94	0.04	1.17	0.10
RPS9A1KVC46	1.00	0.11	1.10	0.13	0.79	0.17	0.69	0.15	1.01	0.32
rRED1	1.00	0.35	0.76	0.09	0.84	0.17	0.71	0.13	1.20	0.18
rTGF_beta	1.00	0.03	1.10	0.04	1.09	0.17	1.36	0.10	1.37	0.05
rTP1	1.00	0.28	1.20	0.28	1.06	0.72	1.23	0.57	0.78	0.27
rXMAP215	1.00	0.37	0.66	0.06	0.95	0.06	0.93	0.09	1.74	0.84
SLM2	1.00	0.13	0.83	0.03	0.80	0.07	0.79	0.09	1.02	0.17
SRPSK	1.00	0.17	1.68	0.21	1.84	0.44	3.12	1.40	1.67	0.12
Synapsin1	1.00	0.14	0.89	0.15	1.05	0.30	0.77	0.06	1.36	0.58
Synapsin2	1.00	0.37	1.12	0.21	0.92	0.15	1.55	0.37	1.33	0.70
Synapsin3	1.00	0.21	0.88	0.21	0.67	0.10	0.67	0.34	1.02	0.35
synaptopodin	1.00	0.11	1.33	0.10	1.63	0.28	2.49	0.61	2.75	1.73
TNF-alpha	1.00	0.04	1.06	0.10	2.72	1.03	3.95	1.33	2.64	0.23
tPA	1.00	0.07	1.17	0.14	0.93	0.34	1.23	0.16	1.39	0.06
Transthyretin	1.00	0.80	8.72	3.31	0.00	0.00	2.04	0.70	3.02	1.11
Ubinuclein	1.00	0.05	0.96	0.03	0.79	0.03	0.90	0.07	1.01	0.04
XIACT18A2KVC45	1.00	0.08	0.84	0.18	0.91	0.25	1.04	0.27	0.95	0.20

Table S2

Table S2. List of primers used for real time RT-PCR

Primer set #	*Gene name/Identifier/Acc #*	*sense primer*	*anti-sense primer*
1	ADT2	GGGTGGTGCCTTTGTGCTTGT	TCTCGGGGAGAACACCTAGGACA
2	Alpha-spectrin 2	GGCTGCCCTCATTCCCACTTAA	TGGGAAGGAAACAGGGGGACTA
3	Ania3	TTGGCACATGTAACTGCCCAAGT	AGGTAGGGCGGAGGATTCATGA
4	APPBP2	CAGGAATCCCCACTATCGTGCC	TGGGGATGGTAGGAAAGGCTGT
5	Arc	AGGAGAGCTGCCTGAGCAGG	TGCTCCAGGGTCTTGGGAAGT
6	Arcadlin	GTGGAAGTTAACTGCCAAAGGAATT	TCCCATTAAGCAATTCCAACATGT
7	ATPsynthase6	GCCGTGATTGTAGTCTTCCTGCT	CAGAGGCTCTTGGGATGAAATCA
8	bad2	CCATGGGAGGAGCAATGCAATA	GGGGGGGGGGGTGTTAAATAA
9	bdnf	ATGAAGGCTGCGCCCATGA	CTGCCCTGGGCCCATTCA
10	beta_actin	TCTGTGTGGATTGGTGGCTCTA	CTGCTTGCTGATCCACATCTG
11	BI303981	CAGAGTCGCTACAGGCAGATGG	GATGGGCTGGCGGCAAAT
12	c-fos	AAATCAAAAGCAACCGCATGGA	CAGGCCTGGCTCACATGCTACT
13	C/EBPb	GCCCGCCGCCTTTAGACC	CCAGGCAGTCGGGCTCGTAG
14	Cdk5	ATCCATCCTTCCGTGCAGTTATT	GCAGGGGATGGCACATTTTCT
15	CGI-12	TCTTTCCACGGAGAAGCTCAACA	TTGCCTGTATGGTCAAAAATGCA
16	cGolgip28	CACGATCAACTGAAGCAGCAACA	TGGTTTGTTGTTGGAGGGTTCA
17	CO I	CCCGAGCCTACTTTACATCTGCC	CCCTAAGGCTCATAATATGGCGG
18	CO II	CGAAGACGTCCTGCACTCATGA	CCGCAAATTTCAGAGCATTGG
19	COX2	CAACCTGCTGACTGAACCTGGG	GAGGCACGGCAGCAGTCACA
20	CRBP2C2KVC60	GCAAGGCAAGTTCAAACCCAACA	TGGCCTCCCTGTTAATGGTTGA
21	CyB	TTTCATCAGTCACCCACATCT	ATGGGTGTTCTACTGGTTGG
22	cyclophilin	TGTGCCAGGGTGGTGACTT	TCAAATTTCTCTCCGTAGATGGACTT
23	CystatinC	GTACCACAGCCGCGCCATACA	CGGCCCATCTCCACATCCAAAT
24	DAPIT	AGTCATGGCTGGCCCAGAAAGT	GACCAACAAAGCAATGCCTCCA
25	DDX1	TCATACACCTTGGCTACCTTCCC	TCAGAAGCACCACCACGTTAAGA
26	Dendrin	TCGCAGGGTTGTTGTGAGGATTT	CAGGCACTGTGCAAGACGCTTTA
27	DRP2	CTTCTTTCTTTTTCAAATTT	TCCGTGGTCATATAACACAG
28	Egr3	AGGGAGACGTGGAGGCCATGT	GAGGTCGCCGCAGTTGGAATA
29	EST00098	GGCACGGAAGGCGAGAATGA	CAACATGGAGTTGATGGCAGCA
30	FGF-2	ATGGACGGCTGCTGGCTTCT	TTGGACTCCAGGCGTTCAAAGA
31	FLJ37318	CAGCTCCTCTTCCCTCCCTGTC	GGGCATCAGGACATCTGGGGA
32	GABAT	GAGCAGGTGGCTGGCCTTTG	CCATCACCCTCTCTGGCAGCAT
33	GCbGT	CTTGGCTTGGAATGCACTCACC	GATGGAGCCCACGCTGTAAACA
34	GDNF	TGACTTGGGTTTGGGCTACGAA	GTACATTGTCTCGGCCGCTTCA
35	Glypican4	ATCACCAATACACAACTAAC	TTCAAGTGTGTCCCTCCGCA
36	GST3-DD4G1KVC38	TGACAGGCTACACACTCTACT	GCATTTGATGGTTCTATACC
37	H-ATPase	TCTTGTGGCACGTGTAGCAATGT	CGGCAGAGGCACACAGAATACA
38	homer1a	CCTGCTCCAAAGGAAAGCCTTG	ATGCTGACGGGTTCCTGCCT
39	homer1b/c	GGAGCAGCGCCTGGAGAAGA	AGGTTACTGCGGAAAGCGTCCT
40	HSP70-B1A1KVC49	CGCTTTGATTACCCCGATTGA	TTGCTTTCCCCAAATATCCATCA
41	IL-1alpha	AAATCCTCTGAGCTTGCCAGGC	GAGCTCCACGGATGTGGAAACA
42	IL-6	TCTCCGCAAGAGACTTCCAGCC	GGAAGGCAGTGGCTGTCAACAA
43	KIAA0833	TCTTTGTTGGTGTGAGTGGATGG	GGTGCCAAGTAATGTGAATGCTG
44	KID1	GGGCCTGTTTCCTTTGCTTTGA	CTTAGCCCGCAGCCATCTTTG
45	Kif21a	TTTGTGCCCGTTGGAGAGATGA	TCCAGATTCTCACGGTTCGATCA
46	krox20	CTGCGAGCCCTTCCCTTTGA	CCCAAGCCATTAAAGTGCCACA
47	krox24	GCCACCACCTATGCCTCCGT	CCCGTTGAGGTGCTGAAGGAGT
48	TIEG1	GATGGCTTATGTCATCAGCAAAGGA	CACCTGTCCATCTGTGATGGTCACT
49	mGluR4	CGCCTTGCCCTTCCTGCTAAC	CCATCACCAAACACCCAGGCA
50	mGluR7	ATGCCCCCCTATGACTGACCAA	CAGAACTGGAAGAGAATGGAAAGGG
51	MIF2	GCACAGTTGGAAATGGAGGATGA	ACGCCTCCTGTCTGCTGCTG
52	mit.F1-ATPase	CGGATGCAAAGCTGAAGGAAAT	GGTTCAAACCCAGCCAAGAAGT
53	mLIM13C1KVC56	GTGGGTTCGCTGTGCCTATCA	GATCTGGCACCGTAAAGGAAACA
54	mLIM18G1KVC32	AGGGTTTACGACCTCGATGTTGG	TGCTCCGGTCTGAACTCAGATCA
55	MMP-2	GGACCCTGAAACCGTGGATGA	CGCAGCGGAGTGACGTCG
56	MMP-9	ATGGCAACGGAGACGGCAA	GGTGGCGCACCAGCGATAA
57	MSS4	CCCAACACAGGCGTGCTTTAA	CCGCGGCCGTTTAACTTTAGT
58	mtGPDH	TTGTGCTACTGTTCCGCTTATGC	CCCAGAATGAATGAGGCAGAAAG
59	mVIMC2KVC54	CTGTGGTGTGCGTGTGCTTCTG	CACACGCGCACACAGACACG
60	MyoVa	CACCCTGAACAACCTCCTGCTG	GCCTTTGCTCCAGGAGCACAT

Table S2 (continued)

Table S2. List of primers used for real time RT-PCR (continued)

Primer set #	*Gene name/Identifier/Acc #*	*sense primer*	*anti-sense primer*
61	Narp	CGCCAACTGCTCCACGAACA	TTGTTGTCCACCCATGGATGA
62	NCS1	ACACCGTGGAGCTCCCAGAAGA	GCCTTGGAGCCTTCCTGGAACT
63	Neuregulin	CATCTGTATCGCCCTGCTGGTG	CTGCCGCTGCTTCTTGGTTTT
64	Neuritin	CCGTGAGAGCAGCAGGCAAGT	TCCAGGCCCTGCGGGTAGT
65	ngf	TAAGACCACAGCCACGGACATCA	ATGTTCACCTCGCCCAGCACT
66	NPY	GGCTGTGTGGACTGACCCTCG	CCAGAATGCCCAAACACACGAG
67	NSF	CAGTGACCAAGGGAAACGACCA	TGAGTGTGGAGAGGGAGGTGGA
68	NT-3	CCCAAAGCAGAGGCACCCA	TGCAATCATCGGCTGGAATTCT
69	NT-4	AGTCCTATGTGCGGGCGTTGA	AATCCAGCGCCAGCCCAC
70	PCPTP1	TACTAAGAAGGGATTTTTGA	CATGCGTGCATGACGGTGTC
71	PCTAIRE2	CCACTGCTGCTTTTCACGATTGT	CAGTTAGGCTACGGGGCTTAACA
72	Pim1	GGGCTTCTCGGGCGTCATTA	GCTCCTCGCTCGGTGATGAAGT
73	RACK1	CATCATCAATGCCTTGTGCTTCA	CAATAGTCACCTGCCATACACGC
74	RAP140	ATTGTGAAGCGAGCGCTCCTATT	GCTGAATTGCTGCTCTCTCCTTC
75	rb-A activin	GAAAGAGGTGGATGGAGACGGG	CAGCCCTCCGTCACTTCCGT
76	RB3	GCCTCTGCCTGGACATTCGG	CACCAGGACTCGGCCCTTCA
77	RGS2	AGAGCAGAGGGAGGGAACAGGG	CCTGCTTTCCTGACAGGGACG
78	Rheb	AAAGATTGACGGAGCGGCTTCA	CGTCACATCACCGAGCACGAAG
79	rKCNQ1	CCCATCCAGGTGATGCCAAAT	CCCTGCTGGGCTCTGGATTC
80	rKCNQ2	AGCTGGAGTTCCAGGGCCTTTC	TCCACATTGACCCTGAGAGCGA
81	rKCNQ3	TGTGTCAAGCGCATGCATGTG	TGTGTCAAGCGCATGCATGTG
82	rKCNQ4	GGTAGCTCTTCTCCTCGGCCAC	CTCGCTGCTCTGCTGAGGAGG
83	rKCNQ5	GCTCTCGAGGCAGCCAAGATTT	GGCACCGACCTCCTCATCAGTT
84	rMAP1b	AGAAGGAGCCTATTGCTGCTTGG	GCTCTGTGGTCTTGCCTTCCTTT
85	GAPDH	TGCACCACCAACTGCTTAG	GGATGCAGGGATGATGTT
86	RPL13	TCTTCGGCATCCGAGCAAAGA	CCAGCCGCGCATTATTTCTTCT
87	RPL19	TGAAGGTCAAAGGGAATGTGTTCA	TCTGCCTTCAGTTTGGGGATGT
88	RPL35	ACCTGCGACCCAAGAAGACAAGA	CGCTGCTGCTTCTTGGTCTTCA
89	RPL41	GAGCCAGTGCCGCACCAACT	GCAGTAGGCCCGGGTAAGAACA
90	RPS9A1KVC46	GATGGGATTCCAGAGCCTTCG	GCCTTGGCTAAATCGGTCCC
91	rRED1	GAGCCTTGCGTCTGACCTGG	GCCCAGCACCCTACACATCC
92	rTGF_beta	GTGGACCGCAACAACGCAATCT	CGGGACAGCAATGGGGGTTCT
93	rTP1	AAGCGGAAACCATGTAAGGTGTG	GCACACAACTGCAGCTGCTGTAA
94	rXMAP215	TGTTTGTTGAGATGCTGGATGATTT	GCCACAATTAGGACATCGAAAAGG
95	SLM2	AGCACAAAGCGGAGCGGATTA	GCCGAAGGAGCCTTATGTCTTGA
96	SRPSK	TTGCCTTTTCATTACCGCCCA	CGCTCTGCAAAGGGGAAATGA
97	Synapsin1	ACAGGCTACCCGTCAGGCATCT	CCTGAGACCTTCGGTGGAGCTG
98	Synapsin2	ATCGCCATGTCAGACCGGTACA	TCTTTGCCATGCACAGCTTTGA
99	Synapsin 3	GGAGGACAGGAGGGCAAGAAGA	AAGGAGGGAACCAGACAGGGCT
100	synaptopodin	CAACCAACCCACCGCTGATGT	ATCAAGCGTGCCATTCGGG
101	TNF-alpha	CCCACACCGTCAGCCGATTT	GGCGGAGAGGAGGCTGACTTT
102	tPA	CCACAGCCCCCTAAAACCCTTGA	GTTGAGGATTGTGGGAGGATGGG
103	Transthyretin	GCTTCCCTTCGCCTGTTCCTC	TCGGACAGCATCCAGGACTTTG
104	Ubinuclein	AAAGAAAGGCATAAGGTGGAGGC	CCGATTCATCGTAGCCATACCC
105	XIACT18A2KVC45	TGTGTGCTGTGTCTTCCTGTGCC	CCACTCCTGGGCTCTTTGGG

Table S3

Table S3. Gene-specific primers used for quantitative RT-PCR

Gene	Sense primer[§]	Anti-sense primer[§]
Arc	AGGAGAGCTGCCTGAGCAGG	TGCTCCAGGGTCTTGGGAAGT
Egr3	AGGGAGACGTGGAGGCCATGT	GAGGTCGCCGCAGTTGGAATA
Homer-1a	CCTGCTCCAAAGGAAAGCCTTG	ATGCTGACGGGTTCCTGCCT
Ania-3	TTGGCACATGTAACTGCCCAAGT	AGGTAGGGCGGAGGATTCATGA
MMP-9	ATGGCAACGGAGACGGCAA	GGTGGCGCACCAGCGATAA
Narp	CGCCAACTGCTCCACGAACA	TTGTTGTCCACCCATGGGATGA
c-fos	AAATCAAAAGCAACCGCATGGA	CAGGCCTGGCTCACATGCTACT
NGF	TAAGACCACAGCCACGGACATCA	ATGTTCACCTCGCCCAGCACT
BDNF	ATGAAGGCTGCGCCCATGA	CTGCCCTGGGCCCATTCA
Synaptopodin	CAACCAACCCACCGCTGATGT	ATCAAGCGTGCCATTCGGG
Pim1 kinase	GGGCTTCTCGGGCGTCATTA	GCTCCTCGCTCGGTGATGAAGT
TNF-α	CCCACACCGTCAGCCGATTT	GGCGGAGAGGAGGCTGACTTT
NT-3	CCCAAAGCAGAGGCACCCA	TGCAATCATCGGCTGGAATTCT
RGS2	AGAGCAGAGGGAGGGAACAGGG	CCTGCTTTCCTGACAGGGACG
krox-20	CTGCGAGCCCTTCCCTTTGA	CCCAAGCCATTAAAGTGCCACA
Protocadherin-8	GTGGAAGTTAACTGCCAAAGGAATT	TCCCATTAAGCAATTCCAACATGT
TIEG1	GATGGCTTATGTCATCAGCAAAGGA	CACCTGTCCATCTGTGATGGTCACT
β-A Activin	GAAAGAGGTGGATGGAGACGGG	CAGCCCTCCGTCACTTCCGT
Cox-2	CAACCTGCTGACTGAACCTGGG	GAGGCACGGCAGCAGTCACA
SV2A	AGGGGTGGGGACGACTGTTTC	TGTCCATGAGCAGGGCAGAAAT
GAPDH	TGCACCACCAACTGCTTAG	GGATGCAGGGATGATGTT
β-actin	TCTGTGTGGATTGGTGGCTCTA	CTGCTTGCTGATCCACATCTG
cyclophilin A	TGTGCCAGGGTGGTGACTT	TCAAATTTCTCTCCGTAGATGGACTT
Homer-1b/c	GGAGCAGCGCCTGGAGAAGA	AGGTTACTGCGGAAAGCGTCCT
CAMKII	GCAGACTTTGGCCTGGCCATAG	AGGTCCACAGGCTTCCCGTATG
GluR2	GCCAGAGTCCGGAAATCCAAAG	CGATGCCGTAGCCTTTGGAATC
Synapsin I	ACAGGCTACCCGTCAGGCATCT	CCTGAGACCTTCGGTGGAGCTG
Synaptophysin	CAGGCGCCCCGGAAAAG	TGGCTGGCTGCCCGTAATC

§ primers are in the 5'-3' direction

Figure S1

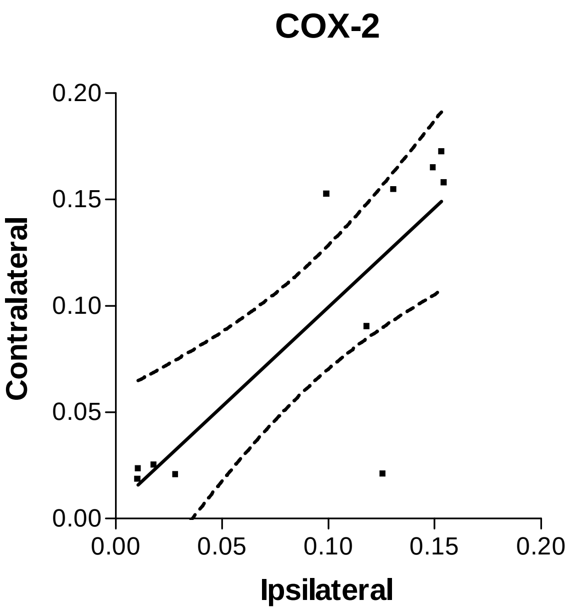

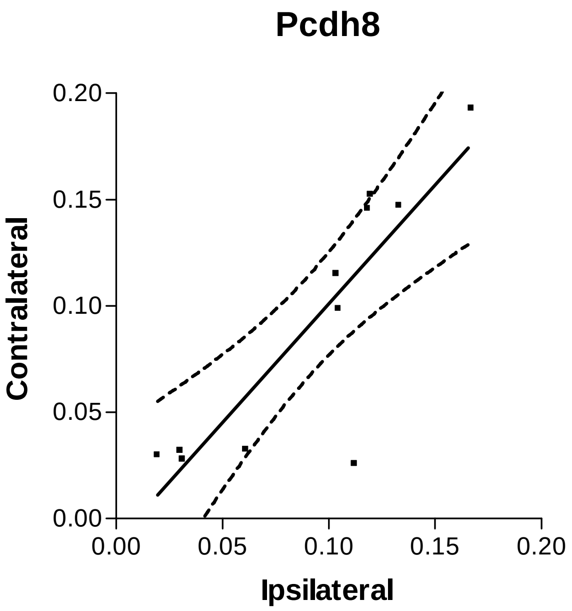

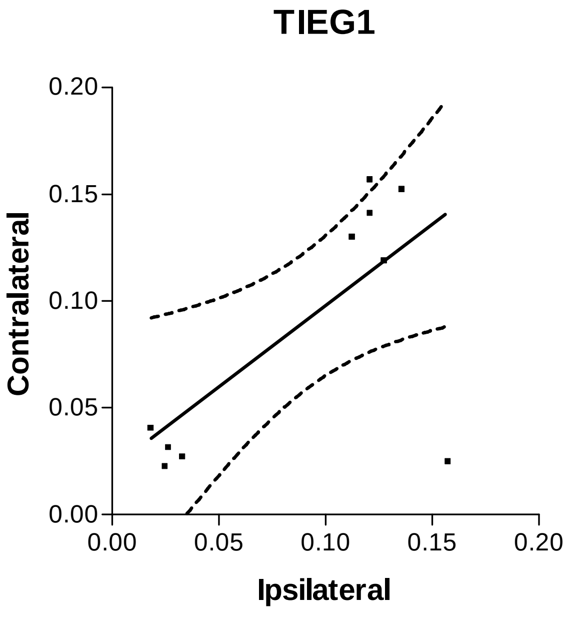

Figure S1. Kindling-induced IEG expression levels in the contralateral hippocampus correlates with IEG expression levels in the ipsilateral hippocampus of amygdala-kindled rats.

Linear regression analysis of ipsi- and contralateral expression levels of transcripts encoding *A*. COX-2, *B*. Pcdh-8 and C.TIEG1 in the hippocampus of 11 individual animals. Quantitative RT-PCR data is presented as relative means of triplicate measurements normalized to the cumulative expression. The correlation coefficient is 0.82 for COX-2 (r2 = 0.6702, P< 0.005), 0.85 for Pcdh-8 (r2 = 0.7251, P<0.001) and 0.67 for TIEG1 (r2 = 0.4469, P<0.05). Dotted lines indicate the 95% confidence intervals.

Figure S2

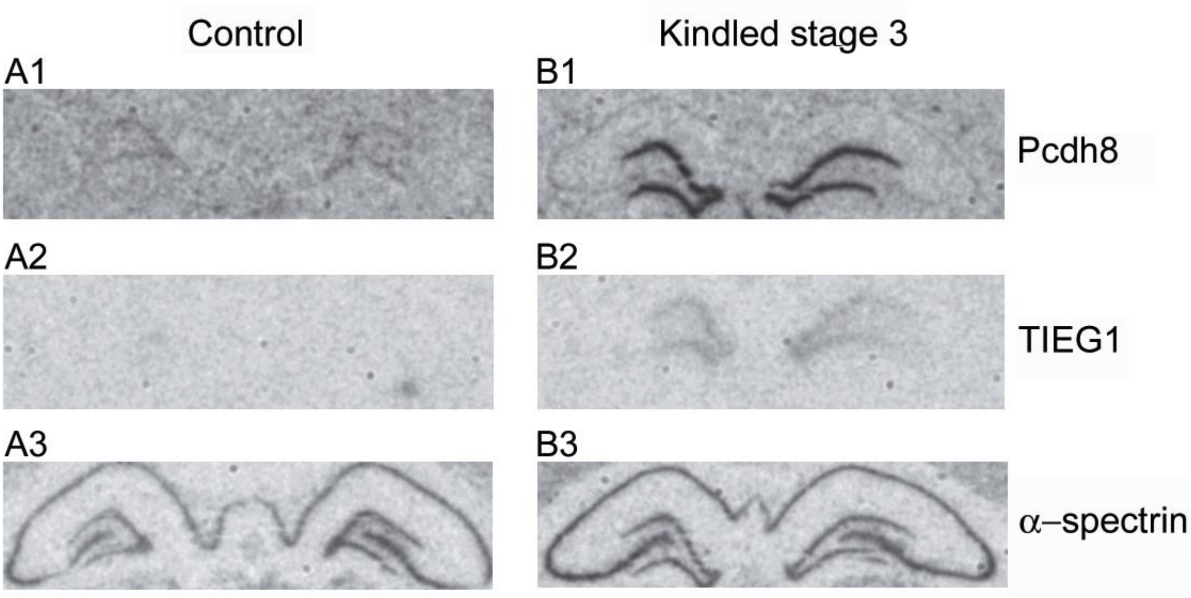

Figure S2. The differential expression of the Pcdh-8 and TIEG1 transcripts in the hippocampus of amygdala-kindled rats is localized to the dentate gyrus. Autoradiograms of 12 μm cryo sections from *left panel,* a control; and *right panel,* a stage 3 amygdala-kindled rat *in situ* hybridized with 33P-radiolabeled *A1 and B1,* Pcdh-8; *A2* and *B2,* TIEG1; and *A3* and *B3,* α-spectrin anti-sense riboprobes. The increase in expression of TIEG1 and Pcdh-8 in amygdala-kindled rats was almost exclusively seen in the dentate gyrus although faint labeling of Pcdh8 in CA1 and CA3 was also seen (Supplementary Figure S2). The radiolabeling was observed in both the ipsi- and contralateral hippocampus. One of our house-keeping gene controls in the quantitative RT-PCR that showed no differential expression was the transcript encoding α-spectrin (data not shown). Expression of α-spectrin mRNA in the hippocampus of both control and stage 3 kindled rats was determined (Fig. 4A3-B3).

Table S4

Table S4. Kindling-induced IEGs in the ipsilateral hippocampus of amygdala-kindled rats

Functional group	mRNA	Expression level†	Accession #
Transcription factors	Egr3/Pilot	6 up	NM_017086
	c-fos	12 up	NM_022197
	Egr2/krox-20	5 up	NM_053633
Neurotrophic factors	NGF	1.8 up	XM_227525
	BDNF	10 up	NM_012513
	NT-3	1.5 dw	NM_031073
TGF-β superfamily	β-A activin	16 up	NM_017128
	TIEG1	7 up	NM_031135
Inflammatory response	TNF-α	3 up	BC107671
	Cox-2	9 up	NM_017232
GPCR interactions	Homer-1a	13 up	AJ276327
	Ania-3	5 up	AF030088
	RGS-2	2 up	NM_053453
Extracellular proteins	MMP-9	8 up	NM_031055
	Narp	15 up	NM_001034199
Cell adhesion	Protocadherin-8	6 up	NM_022868
Others	Arc	10 up	NM_019361
	Synaptopodin	2.5 up	NM_021695
	Pim-1 kinase	5 up	NM_017034

† normalised expression levels of stage 5 kindling relative to controls

Figure S3

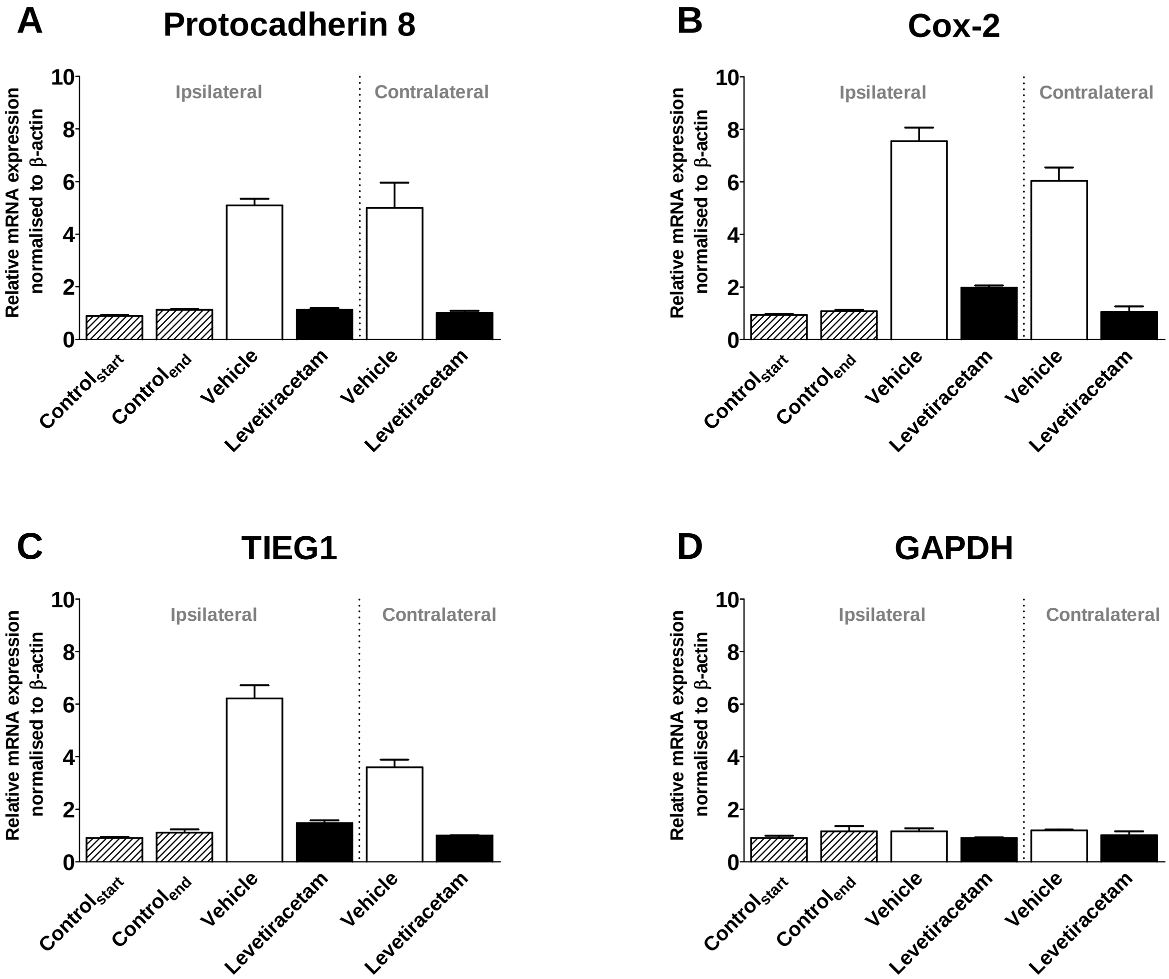

Figure S3. Levetiracetam counteracts the kindling-induced expression of IEGs in the ipsilateral and contralateral hippocampi of amygdala-kindled rats. Quantitative RT-PCR of pooled RNA from the hippocampus of control start ipsilateral (n=5), control end ipsilateral (n=6), ipsilateral vehicle (n=14) and levetiracetam ipsilateral (n=17), contralateral vehicle (n=17) and levetiracetam contralateral (n=17) treated amygdala-kindled rats with gene specific primers directed against *A.* Pcdh-8. *B.* COX-2, *C.* TIEG1 and *D.* GAPDH. The relative expression level of each cDNA are calculated by the ΔΔ-method, normalized to the expression levels of β-actin in the sample, and set relative to the mean normalized expression levels of the control samples. Each quantitative RT-PCR experiment has been done twice of which one representative experiment is shown. Triplicate qPCR data is presented as mean ± SEM.

Figure S4

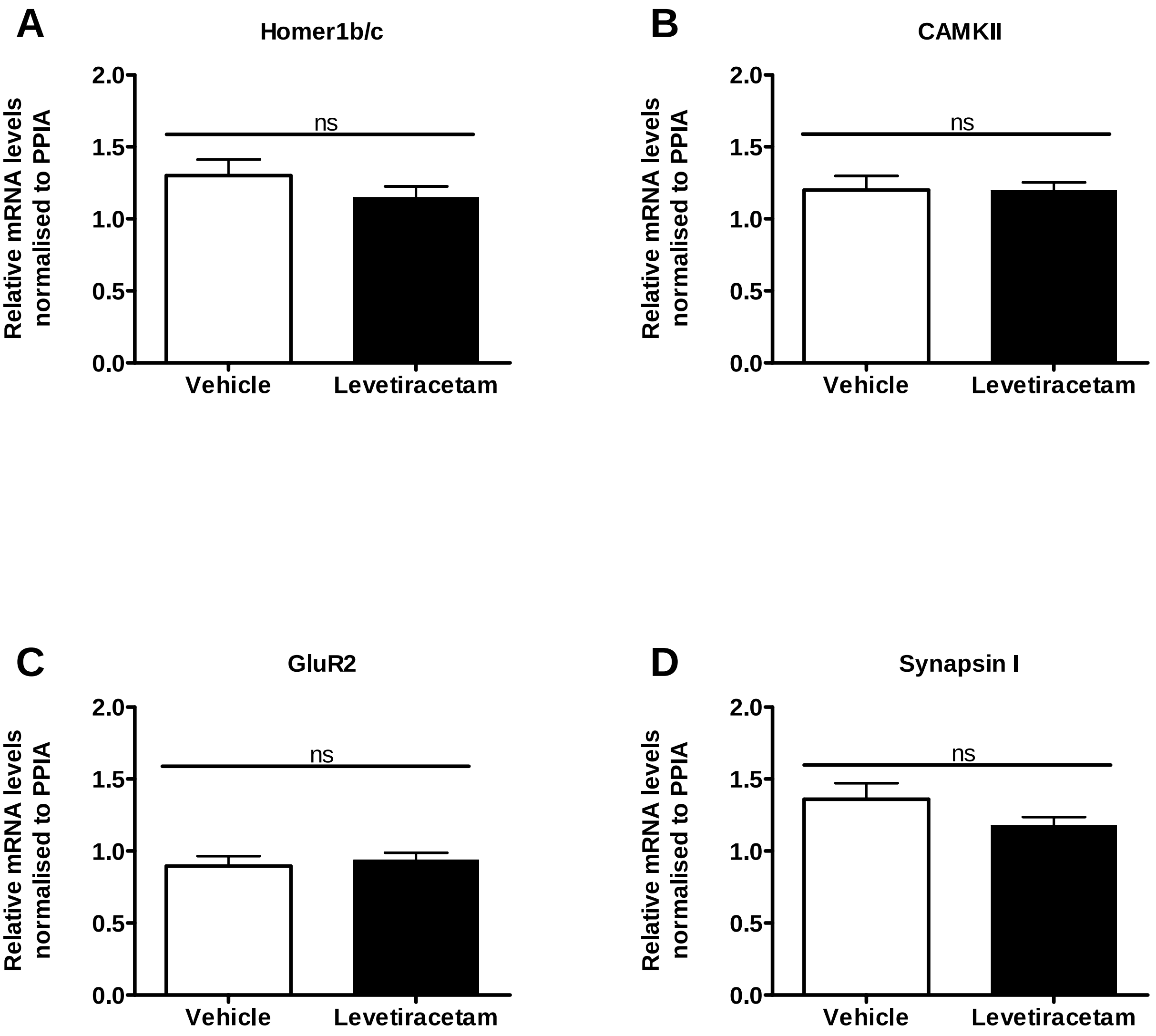

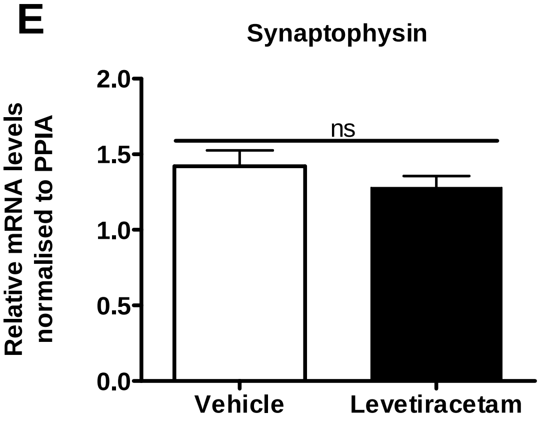

Figure S4. No effect of levetiracetam on constitutively expressed synaptic genes in the contralateral hippocampus of amygdala-kindled rats. Quantitative RT-PCR of total RNA from the hippocampus of contralateral vehicle (n=16) and levetiracetam contralateral (n=16) treated amygdala-kindled rats with gene specific primers directed against *A.* Homer-1b/c. *B.* CAMKII, *C.* GluR2, *D.* Synapsin I and *E*. Synaptophysin. The relative expression level of each cDNA are calculated by the Pfaffl method, normalized to the expression levels of PPIA in the sample, and set relative to the mean normalized expression levels of one of the control samples. qPCR data is presented as mean ± SEM.
